# Resolution of inflammation during multiple sclerosis

**DOI:** 10.1007/s00281-019-00765-0

**Published:** 2019-11-15

**Authors:** F. Ruiz, S. Vigne, C. Pot

**Affiliations:** Laboratories of Neuroimmunology, Neuroscience Research Center and Service of Neurology, Department of Clinical Neurosciences, Lausanne University Hospital and University of Lausanne, Chemin des Boveresses 155, 1066, Epalinges, Switzerland

**Keywords:** Multiple sclerosis, Suppressive immune cells, Innate immune cells, Neurovascular unit, Astrocytes, Blood-brain-barrier, Induction therapies

## Abstract

Multiple sclerosis (MS) is a frequent autoimmune demyelinating disease of the central nervous system (CNS). There are three clinical forms described: relapsing-remitting multiple sclerosis (RRMS), the most common initial presentation (85%) among which, if not treated, about half will transform, into the secondary progressive multiple sclerosis (SPMS) and the primary progressive MS (PPMS) (15%) that is directly progressive without superimposed clinical relapses. Inflammation is present in all subsets of MS. The relapsing/remitting form could represent itself a particular interest for the study of inflammation resolution even though it remains incomplete in MS. Successful resolution of acute inflammation is a highly regulated process and dependent on mechanisms engaged early in the inflammatory response that are scarcely studied in MS. Moreover, recent classes of disease-modifying treatment (DMTs) that are effective against RRMS act by re-establishing the inflammatory imbalance, taking advantage of the pre-existing endogenous suppressor. In this review, we will discuss the active role of regulatory immune cells in inflammation resolution as well as the role of tissue and non-hematopoietic cells as contributors to inflammation resolution. Finally, we will explore how DMTs, more specifically induction therapies, impact the resolution of inflammation during MS.

## Introduction

Multiple sclerosis (MS) is a frequent autoimmune demyelinating disease of the central nervous system (CNS). The exact cause of MS remains elusive but it is certainly a multifactorial disease. Environmental factors, such as Epstein bar virus infection, low vitamin D status, or cigarette smoking contribute to MS development as well as genetic factors, in particular the HLA variant HLA-DRB1*15:01 [1]. The underlying physiopathology of MS is only partially unraveled. Most probably, auto reactive CD4^+^ T cells are activated in the periphery and cross the blood-brain barrier to reach the CNS, known as the “outside-in hypothesis.” Once in the CNS, CD4^+^ T cells are reactivated by local antigen presenting cells, which will trigger an inflammatory reaction, inducing the recruitment of other leukocytes (such as T cells, B cells, and macrophages). A second hypothesis, the “inside-out hypothesis,” suggests that MS is a primary neurodegenerative disease that triggers an autoimmune reaction. We learned from murine models of MS, in particular the experimental autoimmune encephalomyelitis (EAE) and from the treatments that are effective to constrain MS, that the outside-in hypothesis is certainly valid. Peripheral leukocyte trafficking across the blood-brain-barrier is indeed an essential step in the initiation of relapses. The infiltration of pro-inflammatory leukocytes in the CNS further triggers a disruption of the myelin sheath eventually leading to neuronal loss [2]. However, what stimulates the peripheral infiltration of leukocytes into the CNS is still matter of debate.

Predominantly, the disease starts with a relapsing remitting course (RRMS), which may later convert into a secondary progressive disease (SPMS). In a minority of cases, the patients show progression from the onset without superimposed clinical relapses (primary progressive MS, PPMS) [3]. When the disease is progressive, the majority of disease-modifying treatments (DMTs) are inefficient probably because of the compartmentalization of the inflammation in the CNS. RRMS is characterized by flare-ups of neurological symptoms with periods of remissions. The relapses are characterized by an infiltration of peripheral immune cells across the blood-brain barrier (BBB), and blocking leukocyte trafficking from the periphery to the CNS is effective to treat RRMS.

In this review, we will focus on the factors implicated in the resolution of inflammation and discuss how they can be impaired in MS. We will first discuss the immune mechanism involved then the importance of non-immune compartment. Finally, we will briefly explore how disease-modifying treatments impact inflammation resolution.

## Contribution of immune network to MS resolution

Suppressive immune cells, both from the adaptive and innate immunity, prevent exaggerated inflammatory responses. We will first discuss the implication of CD4^+^ T cells, which can be subdivided based on their cytokine profiles in both pro- and anti-inflammatory subsets. Since the original classification by Mosmann and Coffman of CD4^+^ helper T (Th) lymphocytes into Th1 and Th2 subsets [4], their repertoire has expanded: for example, Th17 cells induce immunity against extracellular bacteria and fungi. Exaggerated Th17 response promotes autoimmunity and elevated levels of IL-17 are detected in MS. However, Th17 cells are heterogeneous and under certain conditions, IL-10 secretion renders them non-pathogenic [5]. However, we will here focus on CD4^+^ T regulatory T cell (Tregs) that are well-established players in the resolution of inflammation. Several classes of Tregs are identified: the FoxP3+ regulatory T cells that consist of conventional/natural Treg (nTreg) cells and induced Tregs (iTregs) as well as the type 1 regulatory T (Tr1) cells [6]. We will then discuss the role of CD8^+^ T cells that outnumber CD4^+^ T cells in MS lesions and also contribute to inflammation resolution [7]. In addition, regulatory B cells (Breg) also restrain inflammation. Furthermore, innate immune cells in particular, subsets of NK cells, foamy macrophages as well as myeloid-derived suppressor cells contribute to inflammation resolution during MS [8]. Finally, the implication of pro-resolving lipid mediators (SPMs) in MS resolution will be explored. We will now discuss the implications of each of these immune cells and regulatory mechanisms in more detail.

### Role of FoxP3^+^ regulatory T cells (Tregs)

**Fig. 1:**
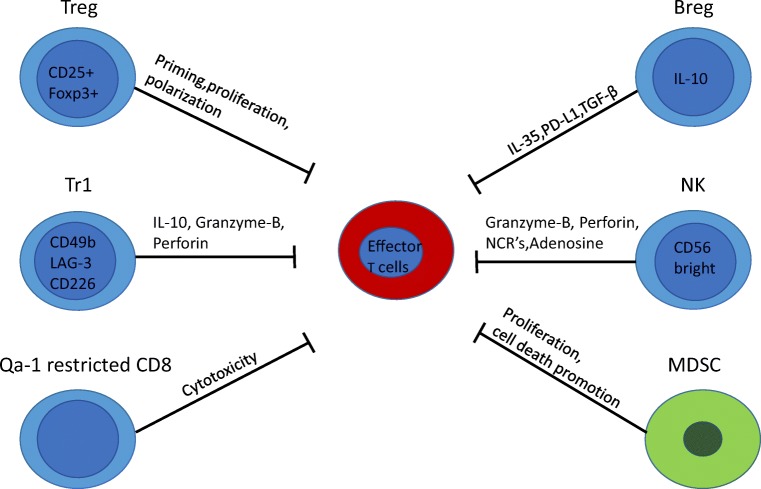
Suppressive immune cells involved in inflammation resolution. Foxp3^+^ Treg cells affect priming, proliferation, and polarization of effector T cells both in the CNS and the periphery. Tr1 cells produce the anti-inflammatory cytokine IL-10 and kill effector cells via granzyme-B and perforin. Qa-1-restricted CD8^+^ cells have a cytotoxic effect on activated CD4^+^ T cells. Bregs secrete IL-35 and TGF-β that suppress APC function. NK cell engagement of NCRs suppresses CD4^+^ T cell proliferation and exerts a cytotoxic activity via the release of granzyme-B, perforin, and of the immunosuppressive adenosine. CNS-derived MDSCs suppress proliferation and promote cell death of lymphocytes

CD4^+^CD25^+^T cells play a critical role in the regulation of CNS autoimmunity in EAE and MS (Fig. 1). Tregs influence EAE by affecting the priming, polarization, and proliferation of effector T cells in the periphery and within the CNS [9]. Transfer of Tregs in the periphery is sufficient to protect mice from the onset and the progression of both active and spontaneous EAE, whereas their depletion exacerbates the disease [10]. In the same line, the presence of myelin proteolipid protein-specific Tregs partially explains the genetic resistance to EAE disease observed in B10.S versus SJL mice. While both mouse models harbor T cells that recognize PLP139-151 at similar frequencies upon immunization, EAE-resistant B10.S mice fail to mount a sustained proliferative response due to a higher relative frequency of PLP-specific Tregs cells in their peripheral repertoire. Indeed, depletion of CD25^+^ cells *in vivo* restores EAE susceptibility to B10.S mice [11]. Furthermore, epigenetic modifications of the Foxp3 gene contribute to the pathogenesis of EAE [12]. Tregs are also implicated directly in the CNS at the site of inflammation resolution and can transmigrate across CNS endothelium [13]. In healthy CNS, Tregs are important in promoting neuroprotection as interactions occur between the resident cells of the CNS and the infiltrating Tregs to modulate the local immune responses [14]. During neuroinflammation, the frequencies of Tregs within the CNS are elevated during the recovery phase of actively induced EAE; however, it is not clearly established if they harbor suppressive activities, at least when tested *ex-vivo* [15]. At the experimental level, treatments that increase Tregs are beneficial; however, current techniques broadly expand polyclonal Tregs but not just Ag-specific cells. Promising studies indicate that tolerogenic nanoparticles induce antigen-specific Tregs and provide protection and transferable tolerance against EAE [16]. In the same way, gene therapy-induced antigen-specific Tregs prevent development and reverses pre-existing EAE [17]. Deficiency and/or dysfunction of Tregs are observed not only in EAE but also in MS [18]. However, whether Foxp3Tregs are deficient in the blood of MS patients has been a matter of debate for several years. To address this question, a meta-analysis regrouping 16 studies was published providing evidences that the proportion of Tregs expressing Foxp3 is indeed decreased in the peripheral blood of MS patients [19]. The assessment of Tregs directly in the CNS is however more challenging. Counter-intuitively, Foxp3+Tregs are increased in the CSF of MS patients; however, their functions are dampened in this compartment [20]. Those results are supported by genome-wide association studies (GWAS) that identified single nucleotide polymorphisms linked to Treg functions associated with an increased risk for MS disease [21].

### Role of IL-10-secreting type 1 regulatory T cells (Tr1)

In contrast to conventional Tregs, Tr1 cells do not express the transcription factor Foxp3 and only a transient expression of CD25; however, they co-expressed CD49b, LAG-3, and CD226 cell-surface markers in humans and mice [22]. C-MAF, AhR, BAFT, and IRF1 are critical transcription factors for Tr1 cell differentiation [23, 24]. Tr1 cells exert their immunosuppressive effects through IL-10 expression and by killing effector cells via Granzyme-B and Perforin (Fig. 1) [6]. Several studies have reported the critical involvement of IL-10 cytokine in the suppression of EAE models associated with an increase in Tr1 cells [25]. In EAE, the transfer of *in vitro* generated OVA-specific Tr1 cells prevents the development of neurological symptoms when OVA peptide is injected intracranially [26]. Moreover, *in vivo* induction of Tr1 cells with soluble myelin basic protein (MBP) reverses ongoing disease in rats immunized with MBP [27]. Regulatory Tr1 cells regulate EAE partially through a mechanism involving IL-10 [28]. Myelin-specific Tr1 cells injected mice-mediated delay onset of EAE associated with a reduction in the severity of clinical signs [29]. Another challenge is to successfully induce regulatory T cells directly *in vivo* to avoid T cell transfer that is challenging in humans. Interestingly, nasal anti-CD3 administration can induce Tr1-like T cells *in vivo* that are further able to constrain inflammation in the progressive animal model of multiple sclerosis in an IL-10-dependent manner by regulating astrocytes and microglia function [30]. Tr1 cells also display immunosuppressive functions in the human setting. Tr1 cells isolated from MS patients display impaired IL-10 production and altered IL-10-mediated suppressive effects when compared with healthy controls [31, 32]. In this line, considering that Tr1 function is impaired in MS, some studies investigated the effect of Tr1 differentiation by tolerogenic dendritic cells (tolDCs). A phase 1b clinical trial showed the feasibility and safety of treating a patient with MS with tolDCs loaded with myelin peptides to increase IL-10 levels in PBMCs as well as the frequency of Tr1 cells [33]. Furthermore, tolDCs exert some of their effects through the cytokine IL-27 that has been identified as a key inducer of Tr1 and inhibitor of Th17 during autoimmunity [6, 24]. IL-27 plays a suppressive role during EAE as demonstrated by more severe disease in IL-27R-deficient mice [34]. Furthermore, IL-27 treatment reduces the severity of EAE by a mechanism dependent on IL-10 [35]. Relevant to the human disease, the beneficial impact of IFNβ, first-line therapy for relapsing-remitting MS, is associated with IL-27 induction, which promotes the production of IL10 by dendritic cells [36]. Finally, IL-27 is expressed by astrocytes in brain biopsies of human MS lesions suggesting that IL-27 regulates T cell response also locally within the brain of MS patients [37]. Therefore, IL-27 (acting on Tr1 cells) plays a critical role in controlling autoimmunity, providing a putative target therapy.

### Role of regulatory CD8 cells

Similarly to CD4^+^ T cells, CD8^+^ T cells are both detrimental and protective during EAE and probably MS. After the identification of CD8 as being protective during EAE [38], the importance of the Qa-1 protein, an MHC class 1b molecule (mouse equivalent of HLA-E for human), as a key molecule in CD8-meditated suppression was highlighted [39]. Qa-1/HLA-E-restricted CD8^+^ Tregs reduce EAE by promoting a cytotoxic activity on activated CD4^+^ T cells (Fig. 1) [40]. By performing screening for TCR specificity, peptides specific for the TCRs of clonally expanded CD8^+^ T cells were identified. Concomitant immunization with myelin and those peptides reduces EAE severity by expanding CD8 T cells that limited the proliferation of myelin-specific CD4^+^ T cells [41]. During an MS exacerbation, CD8^+^ HLA-E-restricted show an impaired cytotoxic activity against activated myelin-specific CD4^+^ T cells [42]. Moreover, neuroantigen-specific CD8^+^ T cells are observed in both MS patients and healthy subjects [43] but their suppressive function is reduced during MS relapses [44]. A meta analysis evaluating the differences of frequency of CD8^+^ Tregs between healthy volunteer and MS patients lead to the conclusion that CD8^+^ Tregs frequency is reduced in MS patients [45]. Furthermore during EAE, while most of the expanded CD4^+^ T cells are myelin specific, the majority of clonally expanded CD8^+^ T cells are not activated by myelin protein [41].

### Role of regulatory B cells

Regulatory B cells (Bregs) represent a small population of B cells, which participates in immunoregulation and suppression of immune responses. Due to the limited data on the phenotype of Bregs, they are usually identified by their capacity to secrete IL-10 and are termed B10 cells (Fig. 1). Apart from their IL-10 production, Bregs exert their functions by the expression of other regulatory cytokines such as TGFβ and IL-35 or by the generation and maintenance of Tregs. The role of B cells itself remained elusive for many years, and initially, it was proposed using B cell-deficient mice, that B cells did not play a major role in the activation of encephalitogenic T cells but may solely partially contribute to the immune modulation in EAE [46]. The roles of B cells were further studied using B cell-targeted monoclonal antibodies (anti-CD20). While B cell depletion is beneficial if performed during disease activity, their depletion prior to EAE induction increases encephalitogenic T cell influx into the CNS in the MOG _35-55_ model of EAE [47]. By using the same strategy, Ray et al. found that B cell depletion prior the onset of EAE resulted in chronic disease induced by adoptive transfer of MBP-specific encephalitogenic T cells [48]. Further studies showed that transfer of B10 cells in mice suppresses active and spontaneous EAE in different mouse strains [49, 50]. The suppressive capacity of Breg during EAE is also dependent on co-inhibitory molecules that downregulates effector T cell responses and elevated PD-L1 expression on B cells suppresses EAE [51]. In addition, IL-35 expression is also implicated in EAE recovery [52]. Finally, the presence of B cell-secreted TGFβ limits the induction phase of EAE, further demonstrating the regulatory role of B cell-derived IL-35 or TGFβ during autoimmunity [53].

In MS pathogenesis, the role of Bregs remains unclear due to several contradictory reports. The number of Bregs was reported to be reduced [54], unaltered [55], or increased [56] ending in disagreement between studies evaluating the role of Breg cells in MS. Interestingly, plasmablasts and plasma cells (that are not targeted by anti-CD20 treatment) highly express IL-10 within MS lesions [57] suggesting that these cells may ameliorate inflammation. IgA+ plasma cells can be generated in the gut and be mobilized to the CNS to further contribute to inflammation resolution in EAE and possibly in MS [58]. The controversies on Bregs are certainly the consequence of variations in patient cohorts (state/form of the disease, treatments) but also due to the non-established phenotype of Bregs and may depend upon the stage of differentiation of B cells.

### Role of NK cells

In addition to regulatory lymphocytes, innate immune cells depict immune-regulatory properties. NK cells are innate lymphocytes that were initially affected with effector function properties, in particular, anti-tumoral and anti-viral [59]. However, they also display regulatory functions, more specifically the subset of human NK cells that express CD56 at high levels (CD56^bright^ NK cells). CD56^bright^ NK cells are a small fraction of circulating NK cells but constitute a large proportion of NK cells within lymph nodes and CSF. NK cells are detected in the CSF of both healthy and MS patients and can be considered as a CNS-specific marker, probably entering the CNS by the lymphatic vessels. NK cells can be activated by the pro-inflammatory cytokines IL-12 and IL-15 and thus are a good prototype of cells induced in the context of inflammation to promote its resolution. Interestingly, IL-27, which drives Tr1 cell generation, further enhances the anti-inflammatory functions of CD56^bright^ NK cells [60]. In human, CD56^bright^ NK cells control T cell responses by several different mechanisms: contact-dependent suppression via perforin and granzyme B, via engagement of natural cytotoxicity receptors (NCRs) or via the secretion of the immunosuppressive molecule adenosine (Fig. 1) [61]. Interestingly, while the numbers of NK cells are similar in control and MS patients, NK cells from MS patients depict a lack of regulatory functions [62]. In EAE, the role of NK cells is more tedious to evaluate, as murine NK cells do not express the surface marker CD56. However, expression of other markers can further identify regulatory murine NK cells. For example, enhancing regulatory NGK2^+^ NK cells dampens EAE disease by killing T and microglia cells in the CNS in the acute phase of EAE. In MS, modulating CD56^bright^ NK cell functions was used as a strategy to tackle inflammatory processes. Indeed, daclizumab, a drug used to treat MS and that blocked the IL-2Rα chain (CD25), was associated with expansion and activation of CD56^bright^ NK cells that further controlled T cell activation [63]. While daclizumab was efficient against MS flare, it was withdrawn from the market after several cases of systemic autoimmunity complications occurred [64].

### Role of foamy macrophages

Histological assessment of the resolving lesion in MS suggests that activated macrophages/microglia play a role in inflammation resolution [65]. Microglia and macrophages can be both detrimental and beneficial during EAE and MS [8]. Of particular interest, foamy macrophage/microglia, which phagocyte myelin, are present in the resolving lesion [65] and MS lesions in general. Foamy macrophages/microglia in MS lesions express anti-inflammatory mediators such as IL-1ra, CCL18, IL-10, TGF-b, and IL-4 [66]. CD163 is considered as a marker of anti-inflammatory M2 macrophages [67], and it was shown that macrophages in acute MS lesions strongly express CD163 [68]. When challenged with LPS, macrophages that have ingested myelin showed reduced production of the pro-inflammatory cytokines TNFα, IL-12p35, and IL-12/23p40 as compared to macrophages that have not ingested myelin [66]. Myelin uptake also increased the production of prostaglandin E2 and CCL18, two mediators that skew macrophages toward an M2 anti-inflammatory phenotype [69, 70]. Myelin ingestion can activate both peroxisome proliferator-activated receptor β/δ (PPAR β/δ) [71] and liver X receptor (LXR) [72]. Those two nuclear receptors are activated by lipids and can repress an inflammatory phenotype [73]. It should be noted that LXR is not a purely anti-inflammatory receptor but can also promote inflammation in certain settings [74].

### Role of myeloid cells

In all MS lesions type, the macrophages/microglia outnumber the lymphocytes [75]. Like all the cells constituting the immune cell network, innate immune cells are both detrimental and beneficial during MS. Among innate immune cells, a potentially interesting subpopulation is the myeloid-derived suppressor cells (MDSCs) that could play an important role in MS (Fig. 1). MDSCs were initially discovered in cancer [76]. They are constituted of heterogeneous populations of immature myeloid cells which have the common ability to suppress T cell proliferation [77]. Their implication in MS is only starting to be investigated and is complicated by the difficulty to define MDSCs (summarized elsewhere [78]). MDSCs accumulate in the CNS during EAE, are able to suppress T cell proliferation, and promote cell death *in vitro* [79]. In addition, the ability of some blood-derived myeloid cells to suppress T cell proliferation was impaired during EAE [80]. Furthermore, those suppressive cells accumulate within the lymphoid compartment during EAE [81] and adoptive transfer of MDSCs attenuates EAE [81]. To corroborate those findings, young mice are resistant to EAE and show a higher frequency of MDSCs [82]. In patients suffering from a MS relapse, the number of circulating MDSCs is increased. Interferon-β, a first-line treatment for MS, could act among many mechanisms by enhancing MDSC activity [83]. Studies are showing both increased and decreased frequencies of MDSCs in MS [81]. More recently, an inverted correlation between MDSCs and CD138^+^ B cells was observed in the CSF of MS patients [84] and CD138+ B cells are positively correlated with CNS inflammation [85]. Based on RNA sequencing analysis, MDSCs could acquire their suppressive phenotype directly in the CNS. They further prevent the accumulation of B cells in the CNS and contribute to dampening the inflammatory reaction [84]. A few publications implicating MDSCs in MS resolution and their potent immune suppressive activity indicates that it is a promising field toward a better understanding of MS resolution.

### Role of TNF and related cytokine

Tumor necrosis factor (TNF) family genes are associated with MS. TNF-α is produced by several cell types including immune cells (macrophages or T cells) as well as CNS-specific cells (astrocytes or neurons) and can be detected in the CNS during MS [86]. It is active under two conformations: a transmembrane protein (tmTNF) and a soluble TNF (solTNF). TNF-α binds to two different receptors with different affinities: TNFR1, expressed on all cell types and TNFR2 mainly expressed on neurons, endothelial and immune cells [87]. SolTNF signals through TNFR1, mediating apoptosis, and chronic inflammation; tmTNF signals by binding both TNFR1 and TNFR2 and promotes resolution of inflammation [88]. TNF-α was initially proposed as a prototypical pro-inflammatory mediator and its expression is associated with MS disease progression [89]. However, blocking TNF-α pathway with lenercept, a recombinant soluble TNFR1 fusion protein strategy, failed as a treatment for MS and even lead to more exacerbations in patients treated with the drug compared to controls [90]. One possible explanation to this phenomenon is the inability of lenercept to enter the CNS [91]. However, treatments with soluble TNFR2 fusion protein (etanercept) or anti-TNF-α antibodies (infliximab) are also associated with the development of MS-like demyelinating lesions in patients treated for rheumatoid arthritis [92] suggesting that TNF-α is possibly actively involved in inflammation resolution and repair processes. First, TNF-α contributes to Treg expansion. Indeed, using *in vitro* co-culture experiments with murine Foxp3^+^ Tregs and effector T cells (Teffs), short-term exposure to TNF-α promotes Teff expansion, and a longer exposition to TNF-α promotes Tregs activation [93]. TNRF2-deficient mice fail to expand Tregs under septic challenge and depict a worse EAE disease course [94]. Furthermore, TNFR2 signaling contributes to tissue repair specifically in the CNS and promotes the proliferation of immature oligodendrocytes [95]. Finally, selective blockade of solTNF improves EAE outcome by enhancing remyelination and axon preservation [95]. In addition, a polymorphism in TNF-related apoptosis-induced ligand (TRAIL) gene, a type II transmembrane protein that can induce apoptosis, is observed in MS [96]. As for TNF-α, the TRAIL/TRAIL-receptor pathway has double roles during neuroinflammation. First, activating TRAIL pathway induces neurotoxicity causing inflammation and cell death [97]. On the other hand, it contributes to inflammation resolution in the CNS as chronic blockade of TRAIL pathway promotes inflammation and demyelination during EAE [98]. In conclusion, the roles of TNF and TNF-related genes on inflammation resolution remain a difficult but exciting area of research in MS.

### Lipid mediators during EAE and multiple sclerosis

Specialized pro-resolving lipid mediators (SPMs) represent promising tools for the treatment of chronic inflammatory diseases. These highly potent anti-inflammatory lipids are derived metabolically from omega-3 essential fatty acids and include lipoxin a4 (LXA4) derived from arachidonic acid (AA), the D-series resolvins, protectins and maresins derived from docosahexaenoic acid (DHA), and the E-series resolvin derived from eicosapentaenoic acid (EPA). They are produced in the resolution phase of acute inflammation and have direct potent cellular responses to dampen inflammation and restore homeostasis [99]. SPMs affect both innate and adaptive immune cells by inhibiting DC maturation and function and/or modulating T and B cell phenotype and cytokine production [99]. Despite emerging data showing that SPMs might control neuroinflammation, research on these mediators in MS remains scarce. However, SPMs are produced in different tissues including the brain and cerebrospinal fluid [100] and accumulative evidence reveal that SPMs reduce neurodegenerative disease and protect neural cells in ischemic stroke or Alzheimer’s disease [101, 102]. Furthermore, a connection between disease severity and lipid mediator production has been proposed as the resolving D1 and the neuroprotection D1 are increased in CSF of patients with highly active MS [103]. In contrast, bioinformatics analysis showed that the metabolites of PUFAs were downregulated in the plasma of EAE, which has also been reported in patients with MS [104]. In addition, oral administration of resolving D1 is effective in attenuating EAE disease progression by promoting Treg phenotype while attenuating the percentage of Th1/Th17 cells [104]. Similarly, the levels of resolving D1 decrease in the CSF of patients with neuromyelitis optica or MS compared to healthy patients, indicating dysfunction of resolution in MS patients [105]. Of note, researches in preclinical models and epidemiologic studies relating specific diets in the management of MS provide preliminary evidence that omega-3 fatty acid supplementation beneficially influences both EAE and MS disease [106]. These observations further strengthen the therapeutic potential of SPMs derived from omega-3 essential fatty acids in the resolution neuroinflammation.

## Contribution of non-hematopoietic component: CNS network

**Fig. 2:**
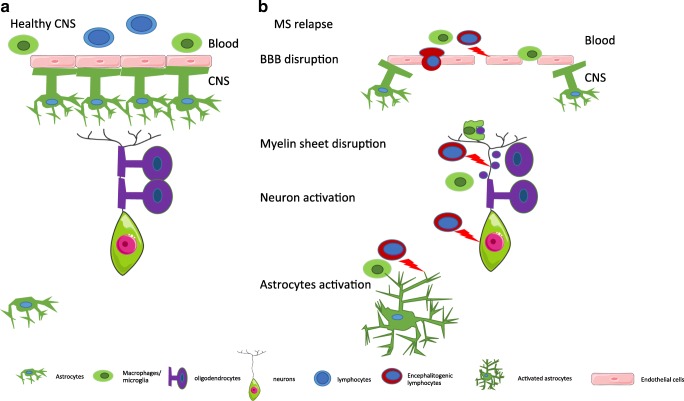
Schematic representation of the CNS at steady-state and during a relapse. **a**. Healthy CNS. The endothelial cells of the BBB are ensheeted by astrocytic end-feet. The BBB is impermeable notably to leukocytes. Oligodendrocytes form the myelin layer that surrounds the axon. **b** CNS during MS. The blood-brain barrier is disrupted and the endothelial permeability is increased. The astrocytic end-feet are detached, allowing leukocytes to transmigrate and trigger an inflammatory cascade. Inflammatory signals produced by leukocytes activate astrocytes. The myelin sheet is disrupted and phagocytes start to remove myelin debris. Neurons are further activated during the inflammatory reaction

The CNS has been considered as an immune-privileged site for decades, based on an original finding that foreign tissue was not rejected when grafted in the CNS parenchyma [107]. The aim here is not to describe the mechanism at the origin of this immune-privilege but to discuss the putative role of non-hematopoietic cells in the resolution of inflammation. The intrinsically immuno-suppressive nature of the CNS is an important concept to consider when evoking local mechanisms that contribute to dampening an inflammatory reaction arising in the brain and spinal cord (Fig. 2a). The infiltration of peripheral leukocytes into the CNS is a key step at the origin of inflammatory cascade leading to MS relapses (Fig. 2b). In-between the blood circulation and the CNS parenchyma lies a “two-wall castle” [108] that needs to be crossed by leukocytes to trigger a relapse, the outer wall being either the highly specialized epithelial cells or endothelial cells respectively of the choroid plexus, i.e., the blood-cerebrospinal fluid barrier (BCSFB) and the CNS capillaries, i.e., blood-brain-barrier (BBB). The inner wall is called the glia limitans as is composed of astrocytic end-feet and the parenchymal basement membrane. In between those “two walls” is found the cerebrospinal fluid (CSF). Virtually, all the cellular networks constituting the CNS mediate pro-resolving mechanisms. The BBB constitutes a tightly regulated obstacle to leukocyte entry in the CNS and in itself can limit the infiltration by counter-regulatory mechanisms. The BCSFB and the CSF serve as selective gateways that shift the infiltrating leukocytes toward a pro-resolving phenotype. The astrocytes, by the production of anti-inflammatory signals and by forming a physical barrier, are key in the MS relapse resolutions (Fig. 2b). Finally, neurons besides their numerous functions are also able to modulate the phenotype of immune cells. The highly specialized tightly sealed endothelial cells of the BBB are an obstacle for inflammatory immune cell trafficking into CNS [109]. However, the BBB endothelium should not be considered as just a simple physical barrier. The regulation of its permeability results from a dynamic cellular cross-talk between endothelial cells, astrocytes, pericytes, microglial cells, and neurons. Together, these cells form the neurovascular unit (NVU) [110].

### Astrocytes

**Fig. 3:**
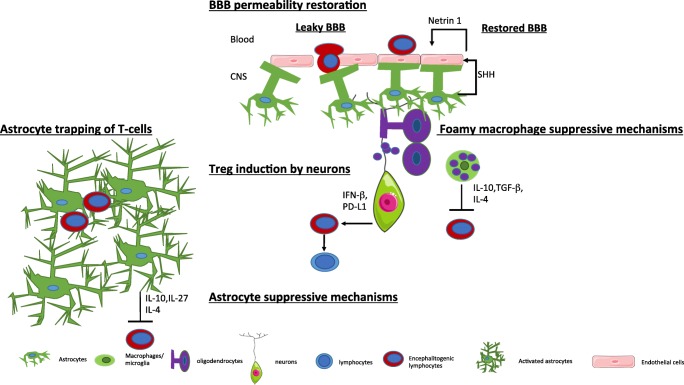
CNS network mechanism promoting resolution. BBB permeability restoration: Shh derived from astrocytes promotes Netrin 1 production by endothelial cells. This pathway reduces the BBB permeability and limits leukocyte infiltration. Astrocytes trap T-cells: activated astrocytes form a physical barrier that limits leucocyte infiltration. Astrocyte suppressive mechanisms: activated astrocytes produce anti-inflammatory cytokines that repress encephalitogenic T cells. Treg induction by neurons: neurons can repolarize encephalitogenic T cell into FoxA1^+^ Tregs. Foamy macrophage-suppressive mechanisms: foamy macrophages produce anti-inflammatory cytokine that contribute to inflammation resolution

The impact of astrocytes in inflammation can be both detrimental and beneficial [111]. However, as the purpose of this review is to give an overview of the potential mechanism resolving an MS relapse, we will focus on protective mechanisms. Astrocytes produce anti-inflammatory cytokines in reaction to an inflammatory stimulus. Activation of human astrocytes by inflammatory cytokines induces the production of IL-27 [112], which is further able to reduce EAE symptoms [34]. In accordance with those results, RRMS patients have increased levels of IL-27 in the CSF [37] and astrocytes show an increased immune-reactivity of the IL-27 subunit EBI3 in MS lesions (Fig. 3) [37]. Furthermore, IL-6-deficient mice are resistant to EAE [113] and IL-6 activation of Gp130 in astrocytes is beneficial during EAE [114]. Using a CRE/Lox system, it has been observed that the specific depletion of gp130 in astrocytes leads to an increased EAE severity associated with a reduced number of FoxP3^+^ CD4^+^ Tregs cells, increased number of IL-17 and IFN-γ producing CD4^+^ T cells. The anti-inflammatory cytokines IL-10 and IL-4 are upregulated in activated astrocytes in MS lesions [115]. Moreover, activation of human-induced pluripotent stem cell-derived astrocytes [116] by a combination of IL-1β and TNF-α induces IL-10 secretion. Finally, the implication of a proliferation of active ligand (APRIL) was explored in MS and EAE: EAE disease is worsened in mice deficient for APRIL. APRIL is expressed in MS lesions, and stimulation of astrocytes with this molecule induces IL-10 secretion that is sufficient to reduce T cell proliferation [117].

We will now explore how the astrocytes-endothelial cellular cross-talk can regulate leukocyte trafficking in MS and EAE. The leukocyte infiltration into the CNS during a relapse is associated with BBB disruption, and thus, restoring or maintaining the endothelial permeability could be a potential mechanism to prevent an excessive infiltration during a relapse. Astrocytes and endothelial cells cross-talk regulate and limit leukocyte infiltration. A key pathway in this cellular cross-talk is the Hedgehog pathway (Hh) [118]. Sonic-hedgehog (Shh) production by astrocytes leads to a decreased endothelial permeability. Blocking the Hh pathways during EAE leads to more severe disease and increases the number of INF-γ and IL-17 producing T cells in the CNS. Finally, increasing amounts of Hh elements are found in MS active lesions. Netrin 1 is produced by the endothelium in response to astrocyte-derived Shh and is upregulated in MS and EAE lesions. Treatment with Netrin 1 can reduce BBB disruption and disease severity during EAE (Fig. 3) [119].

Furthermore, astrocytes limit leukocyte infiltration in the CNS by forming a physical barrier. Any kind of injury in the CNS will lead to the activation of astrocytes, which will be translated into morphological and functional changes called reactive astrogliosis [120]. Reactive astrogliosis can be both beneficial and detrimental. Thus, activated astrocytes attenuate the inflammatory reactions triggered by infiltrating leukocytes by forming a “scar-like perivascular barrier,” a physical barrier that prevents the spread of infiltrating cells to the adjacent CNS [121]. More recently, it has been shown that astrocytes upregulate the tight junction protein CLDN1, CLDN4, and JAM-A when activated [122]. Furthermore, activated astrocytes are able to form interconnected processes leading to a “trap” able to enclose lymphocytes (Fig. 3). This phenomenon is dependent on the expression of CLD1, CLDN4, and JAM-A. Conditional mouse model deficient for CLDN4 in astrocytes leads to increased lesion sizes an increased number of infiltrating CD4 T cells per mm^2^ of lesions during EAE [122].

### Neurons actively participate in inflammation resolution during MS.

The contribution of neurons to the immune privileged site of the CNS is not well established; however, neurons harbor immunoregulatory functions. The interaction between neurons and encephalitogenic T cells induces the conversion of T cells into Foxp3^+^ Tregs (Fig. 3) [123]. A new type of regulatory cells enriched in the CNS of a relapsing-remitting model of EAE was described [124]. The generation of these Tregs cells is dependent on the expression of IFN-β, and their suppressive functions are mediated by the co-inhibitory molecule program death ligand-1 (PDL-1) as well as the expression of the lineage-specification factor FoxA1 (or hepatocytes nuclear factor 3alpha or HNF3 alpha). PDL-1 being mentioned here, we take this opportunity to stress the role of checkpoint inhibitors. Given the increasing number of checkpoint inhibitors such as those that target the PD-1/PDL-1 pathway, notably in the field of oncology, the importance of those inhibitory signals in autoimmunity has been increasingly recognized. Altering checkpoint inhibitory pathways can trigger or worsen CNS autoimmune diseases. For an overview of the implication of those immune-checkpoint and their inhibitors in CNS autoimmune diseases, we refer the reader to the following review [125]. Using a CRE/Lox system, it has been proposed that IFN-β production by neurons is essential to generate FoxA1 Treg cells [126]. Selective depletion of IFN-β expression in neurons leads to an increased number of infiltrating cells in the spinal cord during EAE. The potential implication of neurons as active immune-modulators in MS is an interesting concept that could benefit from deeper investigations.

### Choroid plexus

The epithelial cells of the choroid plexus form the BCSFB. Like the BBB, epithelial cells of the choroid plexus are sealed together with tight junctions [127]. The barrier is thus not formed by the endothelial cells of the choroid plexus which are fenestrated [128]. So far, there is no documentation that crossing the blood-brain barrier could skew leukocytes toward an anti-inflammatory phenotype but the choroid plexus could be considered as a selective gate, facilitating the passage of regulatory cells to the CNS [129]. Moreover, once they have crossed the BCSFB, immune cells reach to cerebrospinal fluid that itself is a suppressive environment [130]. In a model of spinal cord injury, pro-resolving M2 macrophages are reaching the CNS by crossing the BCSFB rather than crossing the BBB at the site of the lesion [131]. The CSF and the choroid plexus are an M2-skewing environment, illustrated by the presence of high levels of IL-13 and TGF-β [131]. However, the implication of the BCSFB as a selective pro-resolving gateway in the context of MS has not been investigated. So far, the evidence suggests that in this context, the choroid plexus rather serve as a primary route of entry for autoreactive lymphocytes [132]. The CCL20 chemokine ligand of CCR6 is constitutively expressed at the choroid plexus [132]. CCR6 is expressed by Th17 cells; it is suggested that during EAE, there are primary wave Th17 cells that reach the CNS by crossing the BCSFB. This primary inflammatory infiltrate would then trigger and the second wave of leukocyte infiltration that would reach the CNS via the BBB in a CCR6-independent manner [132]. It is not excluded that while choroid plexus could serve as an initial route of entry for autoreactive cells in EAE and MS; its pro-resolving nature illustrated in another context could be beneficial during the recovery phase. As Foxp3+Treg also expresses CCR6 [133] during EAE, the choroid plexus could also serve as a site for the recruitment of those cells.

### Endothelial cells in the BBB

Since leukocyte infiltration in the CNS is a tightly regulated process, multiple mechanisms are involved. One treatment strategy to target MS is to inhibit lymphocyte adhesion to the endothelium. Developmental locus-1 (DEL-1) is highly expressed in the CNS [134]. The absence of DEL-1 in the endothelium leads to an increased leukocyte function antigen 1 (LFA-1)-dependent adhesion to the endothelium. Interestingly, mutation in the gene encoding DEL-1 (EDIL3) is associated with MS [135]. DEL-1-deficient-mice display a more severe EAE than their wild-type counterpart [136]. This phenotype is associated with an increased BBB permeability and infiltration of neutrophils together with increased level of IL-17 possibly produced by CD8 T cells. DEL-1 expression is downregulated in chronic active lesions in MS and in EAE [136]. Rather than being a simple adhesion inhibitor, DEL-1 seems to have a pro-resolution function by promoting efferocytosis, a process by which macrophages phagocyte apoptotic neutrophils and acquire a pro-resolution phenotype [137] notably by LXR activation. The implication of this pathway remains to be explored during MS.

## Impact of disease treatment on inflammation resolution

Multiple treatment strategies are available to dampen inflammation in MS. We will here discuss the potential impact of MS treatments on inflammation resolution, first with the treatment of relapse with high-dose of corticosteroids and then novel disease-modifying treatments (DMTs). We will focus on inductive therapies that induce a reset of the immune system and promote component of inflammation resolution.

### Treatment of MS relapse: glucocorticoids

MS course is influenced by the level of endogenous glucocorticoids (Gcs) that are mainly secreted from the adrenal gland in response to an activation of the hypothalamo-pituitary-adrenal (HPA) axis [138]. By interconverting active Gcs (cortisone, 11-dehydrocorticosterone), 11β-HSD modulates intracellular access of glucocorticoid to receptors. Type 1 11β-HSD (11β-HSD1) reactivates glucocorticoids and increases intracellular glucocorticoid concentration while type 2 11β-HSD (11β-HSD2) inactivates Gcs *in vivo*. MS patients show lower cortisol levels in the CSF during acute relapses that may be secondary to poor local activation of cortisone via 11β-HSD1 or to inactivation via 11β-HSD2. In the CNS, differential expression of 11β-HSD1 and 2 expressions in foamy macrophages possibly contribute to the resolution of acute inflammation in MS. The second important “neurosteroids” is the dehydroepiandrosterone (DHEA). In addition to cortisone (the natural metabolite), synthetic steroids are also substrates for the 11β-HSD enzymes. Synthetic Gcs have higher affinity, greater bioavailability, and are poorly metabolized, and thus persist in plasma much longer than endogenous glucocorticoids (cortisol). High-dose corticosteroid medication is thus used to shorten the duration of a relapse and to accelerate its recovery. Corticosteroids have robust anti-inflammatory properties [139], such as induction of T cell apoptosis and inhibition of BBB disruption. They further play an active role in the resolution of inflammation as they increase regulatory T (Treg) number and enhance their suppressive capacities [140]. Indeed, steroid treatment in mice increases the relative number of CD4^+^CD25^+^Treg cells, which are more resistant to GC-induced apoptosis due to a higher expression of Bcl-2 and increased levels of CTLA-4. In humans, the percentage of Treg cells in MS patients is increased after short-term GC therapy [141]. Effects on Treg cells presumably contribute to the therapeutic efficacy of Gcs by favoring active resolution of inflammation during an MS relapse. However, glucocorticoids have no impact on the long-term MS disease course and are not considered as disease-modifying treatment (DMTs).

### Disease-modifying treatments

Disease-modifying treatments (DMTs) are divided in two main approaches: the first DMTs are efficacious through a mechanism of continuous immunosuppression; the second are induction therapies where DMTs reshape the immune system toward a new immune system less prone to disease activity. We will here focus on the second approach, not because it is more commonly used but because of its possible contribution to long-term inflammation resolution. The prototype of induction therapy is the autologous hematopoietic stem cell transplantation (aHSCT) that consists of first mobilization of CD34^+^ hematopoietic stem cells, then an immunoablative conditioning followed by HSC transplantation. aHSCT enables recalibration of the immune system and restores the predominance of anti-inflammatory regulating factors over inflammatory effectors. These mechanisms may explain induction of long-lasting suppression of some autoimmune diseases by aHSCT with the development of a tolerant environment. Tregs are both quantitatively and qualitatively modified in MS patients after aHSCT (review in [142]). The effect of aHSCT on Bregs has not been evaluated in MS but Bregs are increased when aHSCT is performed in patients suffering from systemic sclerosis [143]. Increased PD-1 inhibitory signaling is another possible immunoregulatory mechanism by which aHSCT restores immune tolerance in MS patients with early expansion of PD-1^+^CD8^+^T cells and of PD-1-expressing CD19^+^ B cells. PD-1/PDL pathways play a role in MS and in mouse model of EAE, blockade of PDL1 or PDL2 accelerates disease course and severity [144]. An association between PD-1 deficiency and MS progression is reported: PD-1 expression is higher on MBP-specific CD4^+^ and CD8^+^ T cells during remission compared to acute relapses [145].

In addition, alemtuzumab leads to a “reset of the immune system.” This treatment administered as a pulsed therapy targets CD52^+^ cells and depletes T, B, and NK cells followed by an immune reconstitution. Alemtuzumab increased the anti-inflammatory IL-10 and TGF-β cytokine levels within 6 months of treatment and further increase Treg percentage and function after 24 months post-treatment [146]. However, alemtuzumab increases the percentage of repopulated naïve/immature B cells and possibly the hyper-population of naïve B cell population regenerating before Tregs induce systemic loss of immune-tolerance and secondary autoimmunity that can be severe and limits its use in the clinic [147]. Finally, cladribine can be considered as an inductive therapy. It is a purine analog, a pro-drug whose metabolite selectively accumulates in lymphocytes inducing specific lymphocyte depletion. Cladribine therapy may have a profile of immune-reconstitution closer to aHSCT compared to alemtuzumab [147].

## Open questions and concluding remarks

It is legitimate to argue that an inflammatory misbalance is at the origin of MS. Research on pro-inflammatory mechanisms in MS has been largely studied; however, the potential mechanisms that actively participate in resolving inflammation were scarcely evaluated. In fact, autoimmune diseases in general could come as much from an excess of inflammation as from a deficit of pro-resolutive mechanism or as a combination of both. For example, even if it is purely speculative, the DEL-1 mutation associated with MS [135] could result in a loss of function leading to enhanced leukocyte adhesion and transmigration across the BBB while at the same time, it could contribute to disrupting efferocytosis, an important process for inflammation resolution [148]. In MS, the lack of full resolution of inflammation probably participates to persistent chronic inflammation. For this reason, the comparison of immune responses between MS and other self-limited inflammatory diseases of the CNS like ADEM or viral encephalitis could help in understanding the specific mechanisms that lead to chronic inflammation. A first response to this question was addressed in pediatric neuroinflammatory diseases [149]. By comparing both abnormal effector and regulatory T cells, subsets in children with either MS or monophasic inflammatory CNS disorders, the authors could identify specific abnormalities in MS but not in other self-limited diseases. Only children with MS presented with both an exaggerated pro-inflammatory response of CD8^+^ Teff cells that were resistant to suppression to Tregs as well as deficient suppressive capacities of Tregs (more specifically CD4^+^CD25^hi^CD127^low^Foxp3^+^ Tregs) [149].

Furthermore, the resolution of inflammation is the results of a complex collaboration between the network of peripheral and resident immune cells together with the local cells of the CNS. Along the same lines, it is important to develop new models that specifically mimic inflammatory processes that take place in MS. Interestingly, an alternative rat EAE model that induces focal cortical demyelinating lesions could highlight that rapid resolution of inflammation contributes to more efficient remyelination [150]. A better understanding of this endogenous pro-resolutive mechanism could lead to novel therapeutic approaches. Notably, the use of SPMs for relapse treatment could be a promising new therapeutic approach also in MS. Similarly, favoring astrocytic reaction that prevents the spread of the lesion could reduce the potential disability induced by a relapse. Favoring and accelerating inflammation resolution could be beneficial in terms of preventing the accumulation of disability that we see in RRMS patients.

In conclusion, the understanding of the immunopathogenic mechanisms involved in resolution of inflammation in MS remains an important research field. Thus, a better understanding of resolution of inflammation in MS could result in interesting alternative approaches toward improvement in the treatments of MS patients.
